# Role of IL-1 Beta in the Development of Human T_H_17 Cells: Lesson from *NLPR3* Mutated Patients

**DOI:** 10.1371/journal.pone.0020014

**Published:** 2011-05-26

**Authors:** Denise Lasigliè, Elisabetta Traggiai, Silvia Federici, Maria Alessio, Antonella Buoncompagni, Andrea Accogli, Sabrina Chiesa, Federica Penco, Alberto Martini, Marco Gattorno

**Affiliations:** 1 Rheumatology Unit, Second Division of Pediatrics “G. Gaslini” Institute, Genoa, Italy; 2 Laboratory of Immunology of Rheumatic diseases, Department of Pediatrics, University of Genoa, Genoa, Italy; 3 Department of Pediatrics, Federico II Hospital, Naples, Italy; Massachusetts General Hospital and Harvard Medical School, United States of America

## Abstract

**Background:**

T helper 17 cells (T_H_-17) represent a lineage of effector T cells critical in host defence and autoimmunity. In both mouse and human IL-1β has been indicated as a key cytokine for the commitment to T_H_-17 cells. Cryopyrin-associated periodic syndromes (CAPS) are a group of inflammatory diseases associated with mutations of the NLRP3 gene encoding the inflammasome component cryopyrin. In this work we asked whether the deregulated secretion of IL-1β secondary to mutations characterizing these patients could affect the IL-23/IL-17 axis.

**Methodology/Principal Findings:**

A total of 11 CAPS, 26 systemic onset juvenile idiopathic arthritis (SoJIA) patients and 20 healthy controls were analyzed. Serum levels of IL-17 and IL-6 serum were assessed by ELISA assay. Frequency of T_H_17 cells was quantified upon staphylococcus enterotoxin B (SEB) stimulation. Secretion of IL-1β, IL-23 and IL-6 by monocyte derived dendritic cells (MoDCs), were quantified by ELISA assay. A total of 8 CAPS and 11 SoJIA patients were also analysed before and after treatment with IL-1β blockade. Untreated CAPS patients showed significantly increased IL-17 serum levels as well as a higher frequency of T_H_17 compared to control subjects. On the contrary, SoJIA patients displayed a frequency of T_H_17 similar to normal donors, but were found to have significantly increased serum level of IL-6 when compared to CAPS patients or healthy donors. Remarkably, decreased IL-17 serum levels and T_H_17 frequency were observed in CAPS patients following *in vivo* IL-1β blockade. On the same line, MoDCs from CAPS patients exhibited enhanced secretion of IL-1β and IL-23 upon TLRs stimulation, with a reduction after anti-IL-1 treatment.

**Conclusion/Significance:**

These findings further support the central role of IL-1β in the differentiation of T_H_17 in human inflammatory conditions.

## Introduction

T helper 17 (T_H_17) is a subset of effector CD4^+^ T cells crucial for the response to fungal and extracellular bacteria [1], [2]. They also play a pathogenetic role in several animal models of autoimmune diseases [1], [3], as well as in human chronic inflammation [4]–[5]. IL-17 is known to induce the mobilization, recruitment, and activation of neutrophils [5]. Moreover, it is able to stimulate the expression of several proinflammatory cytokines and chemokines by a broad range of cellular targets, including epithelial cells, endothelial cells and macrophages [5].

Human T_H_-17 cells are defined by the expression of surface markers such as CCR6 and CCR4 [6], CD161 [4] and IL-23R [7], as well as by the production of different proinflammatory cytokines such as IL-17A and IL-17F, IL-21, IL-22, TNF–α, IL-9 , IL-10 and IL-26 [5], [8], [9].

The differentiation of naïve T cells into pro-inflammatory T_H_17 is dependent on the extracellular environment in which T cells are activated.

TGF-β and IL-6 have been indicated as the two key cytokines for the *in vitro* differentiation of murine T_H_17 cells [10]–[12]. However, when the development of T_H_17 cells has been analyzed in humans, a number of evidences showed the predominant role of IL-1β (alone or in synergy with other cytokines, i.e. IL-23 and IL-6) [4], [6], [7], raising the hypothesis that a dichotomy between mice and humans exists [5].

Cryopyrin-associated periodic syndromes (CAPS) are a group of rare inherited inflammatory disorders consisting in familial cold-induced autoinflammatory syndrome (FCAS), Muckle-Wells syndrome (MWS) and chronic infantile neurologic, cutaneous, articular (CINCA) syndrome [also named Neonatal Onset Multi-systemic Inflammatory Disease (NOMID)] [13]. These disorders are associated with *NLRP3* (or *CIAS1*) heterozygous mutations which determines an abnormal *in vitro* secretion of IL-1β by monocytes [14]. The specific inhibition of IL-1β leads to a dramatic normalization of the inflammatory manifestations [15], [16] as well as to a strong *in vitro* reduction of IL-1β secretion [17]. For this reason CAPS represent a suitable model to verify the actual role of IL-1β in the generation of T_H_17 cells in humans. Here we report that exaggerated IL-1β secretion due to *NLPR3* mutations affects the IL-23/IL-17 axis in CAPS patients, providing further evidence of the role of IL-1β in T_H_17 differentiation.

## Methods

A total of 11 CAPS (6 CINCA, 5 MWS) were enrolled in the study (Table 1). Twenty-six systemic onset juvenile idiopathic arthritis (SoJIA) patients with active disease and 20 aged-matched healthy individuals were used as disease-and healthy -controls, respectively. Samples were taken after informed consent approved by “G. Gaslini” Ethical board.

**Table 1:** Clinical characteristics of CAPS and SoJIA patients at the moment of the study.

	Male/Female	Age(years; range)	Disease duration(years; range)	CRP mg/dL(mean, range)	Rash	Active Arthritis	Fever
CAPS(n = 11)	7/4	11.7 (2.6–43)	10.6 (0.6–22.8)	5.2 (1.9–10.2)	10/11	8/11	6/11
SoJIA(n = 26)	16/10	7.6 (1.5–19.3)	3.6 (0.3–10.9)	12.3 (1.3–25)	11/26	23/26	16/26

CAPS patients displayed the following mutations of *NLRP3* gene: T348M (2 patients), D303N (2 patients), E304K (2 patients), M406I, N477K, E525K, I572F, R260W [18]. At the time of the enrolment in the study, all CAPS patients were naïve from anti-IL-1 and displayed an active phase of their disease, in terms of presence of disease-related clinical manifestations and elevation of acute phase reactants. Disease activity in SoJIA patients was defined by the presence of at least two of the main clinical manifestations (fever, arthritis, rash) and elevation of acute phase reactants despite ongoing therapy with non steroidal anti-inflammatory drugs, oral steroids and/or methotrexate. Peripheral blood mononuclear cells (PBMCs) were isolated by density gradient centrifugation using Fycoll from Lympholyte®, Cederlane. Eight CAPS patients and 11 SoJIA patients were evaluated the day before and 7–10 days after anti-IL-1 treatment (Anakinra).

### Flow cytometry

Thawed PBMCs were stained with the following antibodies: allophycocyanin conjugated anti human CD4 (APC Beckton Dickinson, BD), fluorescein isothiocyanate conjugated anti human CD45RA (FITC Beckman Coulter), phycoerythrin conjugated anti human CCR6 (PE, BD) and phycoerythrin conjugated anti human CD161 (Beckman Coulter). Cells were incubated with 50 µl of PBS 1% FCS for 20 min at 4°C in the dark and acquired with a FACSCanto cytometer. Data were analyzed with Flow-Jo software. Quantification of circulating memory CCR6^+^ and CD161^+^ was determined electronically gating on alive lymphocytes and expressed as the absolute number (number×10^3^/mm^3^) multiplying the relative percentage of the different subsets by the peripheral lymphocytes count.

### Quantification of T_H_17 producing cells

Freshly isolated or thawed PBMCs were stimulated overnight with staphylococcus enterotoxin B (SEB, 100 ng/ml, Sigma Aldrich), anti-CD28 (1 mcg/ml, BD), anti-CD49d (1 mcg/ml, BD) Mabs in the presence of Brefeldin A (BFA 1 mcg/ml, Sigma Aldrich), as described previously [19]–[21]. Surface staining of CD3 (PeCy5, BD), CD8 (APC-Cy7, BD) and intracellular staining of IL-17 (eBiosciences) and IFN-γ (BD) were performed according to manifacturer's instructions.

### Generation and stimulation of monocyte-derived DCs [MoDCs]

Peripheral blood CD14^+^ monocytes were positively isolated with CD14-specific micro-beads (Miltenyi Biotech). DCs were obtained from monocytes after culture with granulocyte-macrophage colony-stimulating factor (GM-CSF, 50 ng/ml, Peprotech) and IL-4 (20 ng/ml Peprotech). At day 7, DCs were cultured for additional 48 h in 96-well flat bottom plates with Lypopolisaccaride (LPS, 1 µg/ml, Sigma Aldrich), *Staphylococcus Aureus (S.a* 10^8^/ml, Invivogen) and Zymosan A (50 µg/ml, Sigma Aldrich).

### Serum and supernatant cytokine detection

Levels of IL-1β, IL-23, IL-17 and IL-6 in culture supernatants or sera were quantified by ELISA assay (eBiosciences) according to the manufacturer's instructions.

### Statistical analysis

Differences among groups were evaluated using the non parametric Kruskal-Wallis test. Post-hoc analysis was performed with non-parametric U Mann-Whitney test. Comparison of variables before and after treatment was performed with Wilcoxon test.

## Results

### IL-17 and IL-6 serum levels and ex-vivo phenotype of circulating CCR6^+^ and CD161^+^ memory CD4^+^ T cells

**Figure 1:**
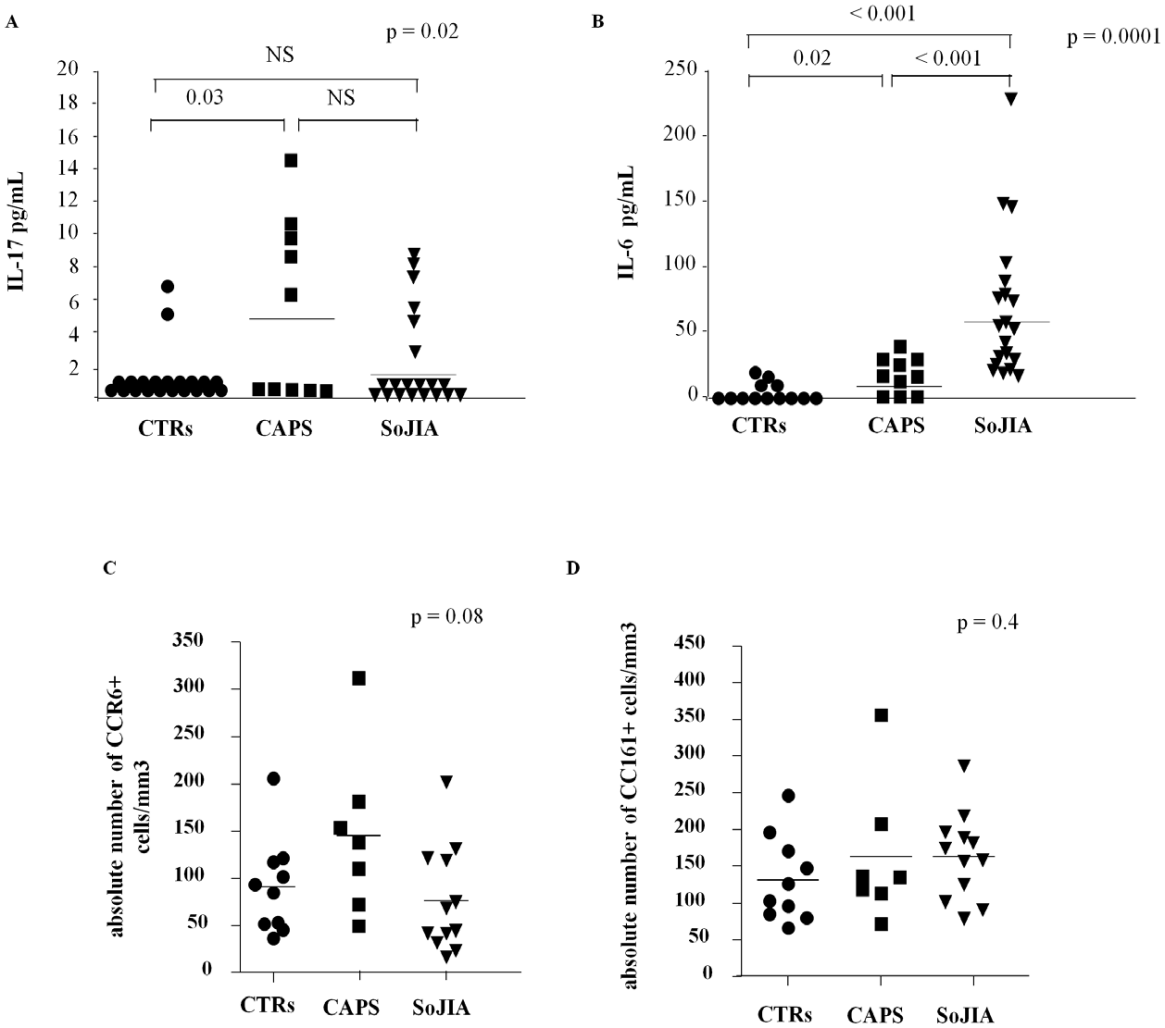
Analysis of IL-17 and IL-6 serum concentrations and peripheral CD4+ T cell phenotype. IL-17 (panel A) and IL-6 (panel B) serum levels were measured in 10 active CAPS patients, 20 active SoJIA patients and 20 healthy controls by ELISA. C–D) *Ex-vivo* analysis of circulating CCR6^+^ (panel B) and CD161^+^ (panel C) memory T cells (CD4^+^CD45RA^−^) in the same three subgroups. Heterogeneity test among groups was evaluated using the non parametric Kruskal-Wallis test (upper right of each graph). Post-hoc analysis with non-parametric U Mann-Whitney test revealed the difference among the three subgroups.

Serum IL-17 was measured in 10 active *NLPR3*-mutated CAPS patients compared to 20 healthy controls and 20 SoJIA patients. As shown in Figure 1A, IL-17 was significantly higher in CAPS patients (median 5,1 pg/ml range:0–14.4) when compared to healthy controls [0.4 pg/ml, 0–6.5 pg/ml) (p = 0.04). A slight elevation of serum IL-17 was also observed in some active SoJIA patients (1.9 pg/ml, 0–8.6 pg/ml), with no statistical differences with either CAPS patients or healthy controls (Figure 1A).

CAPS patients displayed higher serum levels of IL-6 (median 8.4 pg/ml, 0–38 pg/ml) as well when compared to healthy controls (median 2.5 pg/ml, 0–13.5 pg/ml) (P = 0.02). Differently from what observed for IL-17, active SoJIA patients showed a significant elevation of serum IL-6 (median 54.3 pg/ml range:15–230) with respect to CAPS patients and healthy controls (P<0.001) (Figure 1B).

CCR6 and CD161 have been recently indicated as additional markers to define T_H_17 producing cells [22] [4]. Thus, CCR6 and CD161 expression was evaluated in circulating memory CD4^+^ T cells (CD4^+^CD45RA^−^) from 7 *NLPR3*-mutated CAPS patients, 10 healthy controls and 12 active SoJIA. The number of circulating CCR6^+^ memory CD4^+^ T cells in *NLPR3*-mutated CAPS patients (Figure 1C) tent to be higher than that found in the other two subgroups, although the difference was not statistically significant. Conversely, the absolute number of CD161^+^ memory T cells was comparable among the three subgroups (Figure 1D). The same data were observed when the percentage of alive T lymphocytes were evaluated (not shown).

### Frequency of IL-17 and IFN-γ CD4+ T cells in CAPS patients

**Figure 2:**
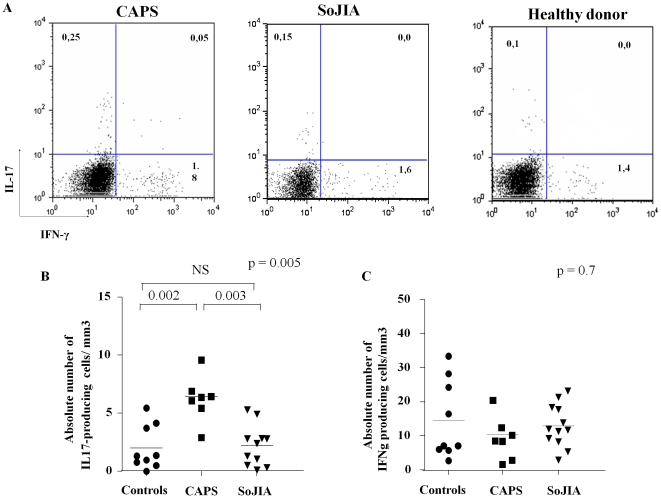
CAPS patients show an higher absolute number of IL-17 producing cells after *in vitro* expansion. A) Dot plot electronically gated on alive CD4^+^ T cells of one representative CINCA patient, one systemic onset juvenile idiopathic arthritis (SoJIA) patient and one healthy control B–C) Absolute number of IL-17 (panel B) and IFN-γ (panel C) producing cells in 7 NLPR3-mutated CAPS and 12 SoJIA active patients and in 9 age-matched healthy controls. Heterogeneity test among groups was evaluated using the non parametric Kruskal-Wallis test (upper right of each graph). Post-hoc analysis with non-parametric U Mann-Whitney test revealed the difference among the three subgroups.

**Figure 3:**
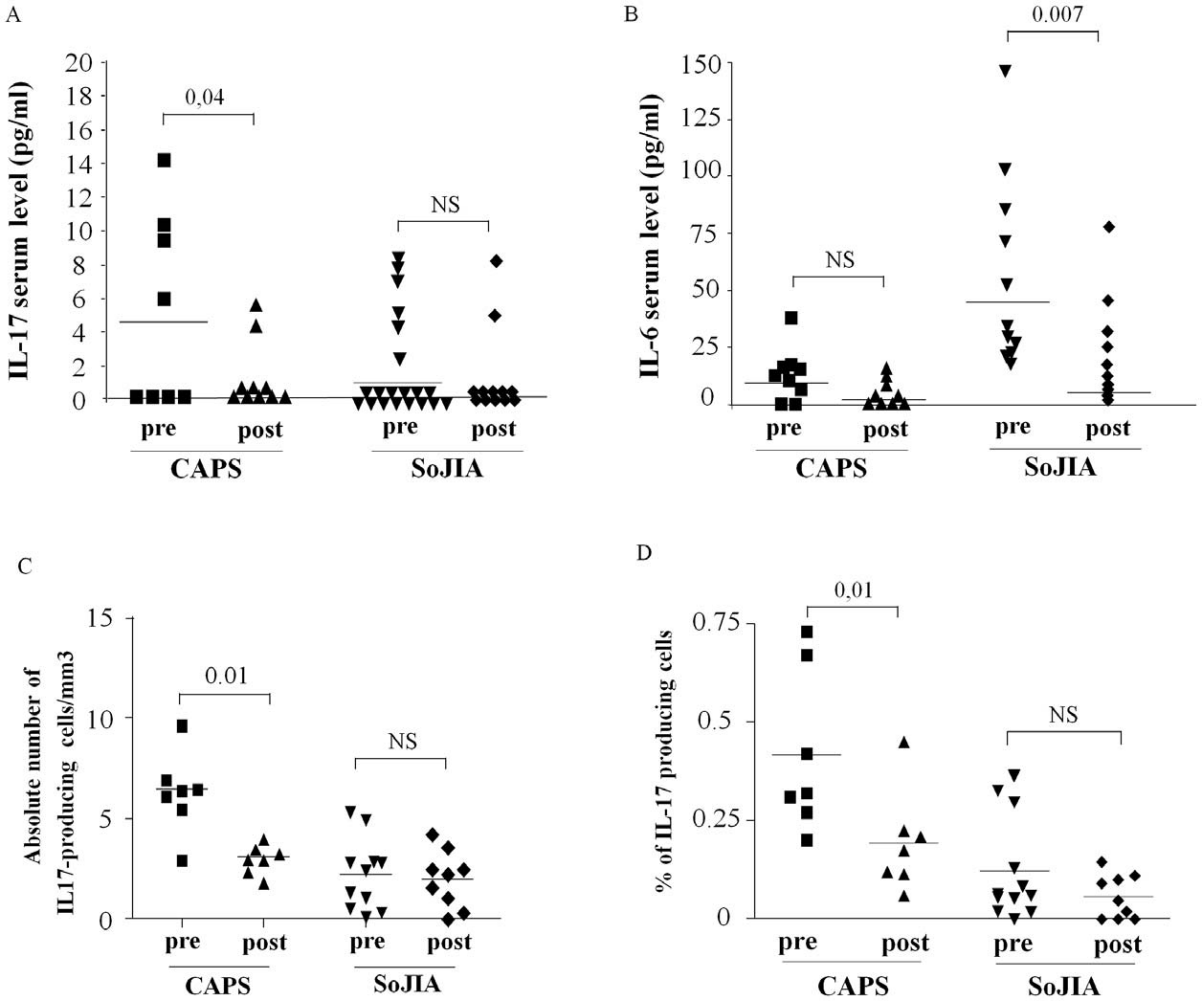
Effect of anti-IL-1 treatment on IL-17 and IL-6 serum levels and Th17 frequency. Variation of IL-17 (panel A) and IL-6 (panel B) serum levels after anti-IL-1 treatment in 8 CAPS patients and 11 SoJIA patients (7 responders and 5 non responders). The decrease frequency of IL-17 producing T cells after anti-IL-1 treatment is reported either as absolute number (Panel C) and percentage of alive lymphocytes (Panel D). Statistical analysis was performed using non-parametric Wilcoxon-Test.

The frequency of IL-17 and IFN-γ producing cells upon anti-CD28, anti-CD49d and SEB stimulation was analyzed according to the experimental protocol recently described by Milner et al. [16]. As shown in Figure 2B, active CAPS patients displayed a significantly higher absolute number (median 7,5 cells/mm^3^, range 2,9–9,5) of IL-17 producing cells compared to healthy controls (median 1,34 cells/mm^3^, range 0,4–5,4) (p = 0.002) and active SoJIA patients (median 1,31 cells/mm^3^, range 0,3–5,2) (p = 0.003). The same results were also obtained when percentage of IL-17 producing cells was assessed (CAPS: median 0,37%, range 0,2–0,73; healthy donors: median 0,1%, range 0–0,28; SoJIA: median 0,06% range 0,0–0,36) (P = 0.01, see also Figure 3D). Conversely, no differences in the absolute numbers (Figure 2C) and percentages (not shown) of IFN-γ producing T cells was observed among the three subgroups.

### Effect of anti-IL-1 treatment on serum IL-17 and frequency of IL-17 producing cells

Most of the CAPS patients analyzed in the present study were treated with IL-1 blockers, namely IL-1 receptor antagonist (Anakinra). As previously shown, the treatment was able to dramatically control the clinical manifestations of all treated patients with a complete normalization of laboratory parameters (hemogram and acute phase reactants) within two weeks [17].

The effect of anti IL-1 treatment on IL-17 serum concentrations and on the frequency of IL-17 producing cells is shown in Figure 3. A significant decrease of IL-17 serum concentration was observed in all CAPS patients after 7–10 days of treatment (Figure 3A). A slight, albeit non significant, decrease was also observed when analyzing IL-6 serum concentration (Figure 3B). Accordingly, treatment with IL-1 blockers was able to normalize the frequency of IL-17 producing cells after SEB stimulation in *NLPR3*-mutated CAPS patients, either when evaluated as absolute number [Figure 3C) or percentage of CD4+ T cells (Figure 3D).

In the present study, 11 SoJIA patients were analyzed before and after anti-IL-1 treatment. After 7–10 days of treatment a significant reduction of IL-6 was observed in anti-IL-1 treated patients (Figure 3B). As previously observed, the long term effect of IL-1 blockade in SoJIA patients is rather variable [23]. In the subsequent follow-up, 7 SoJIA patients displayed a complete response, with a dramatic and persistent control of systemic and articular manifestations, whereas the remaining 5 patients were *incomplete* or *non-responders* [23]. Despite a few SoJIA *responder* patients displayed a decrease serum levels of IL-17 and frequency of IL-17 producing cells after anti-IL-1 treatment, a retrospective evaluation was not able to find any significant differences in the serum levels of IL-17 and IL-6, nor in the frequency of IL-17 producing cells before anti-IL-treatment between *responder* and *non responder* SoJIA patients (data not shown).

### Monocytes-derived dendritic cells from CAPS patients display an increased secretion of IL-1β and IL-23

Since CAPS is associated with a primary defect of the innate immune system, it is conceivable that the T_H_17 skewed phenotype observed in CAPS patients could be related to an aberrant influence on T cell differentiation by antigen-presenting cells rather than by an intrinsic T cells defect [24]. Thus, we analyzed the *in vitro* IL-1β, IL-23 and IL-6 secretion by MoDCs cells after stimulation with TLR ligands in 4 active CAPS patients (2 CINCA and 2 MWS) with *NLPR3* mutation, 4 active SoJIA patients and 9 healthy controls. After stimulation with single TLR-ligand (e.g. LPS), DCs produced variable amount of IL-6 but little of IL-1β and IL-23 either in healthy controls, CAPS and SoJIA patients (data not shown) [25]. Nonetheless, Zymosan, which simultaneously binds to TLR2 dectin-1 and dectin 2 [26]–[27] was able to induce the secretion of detectable amounts of all three cytokines.

**Figure 4:**
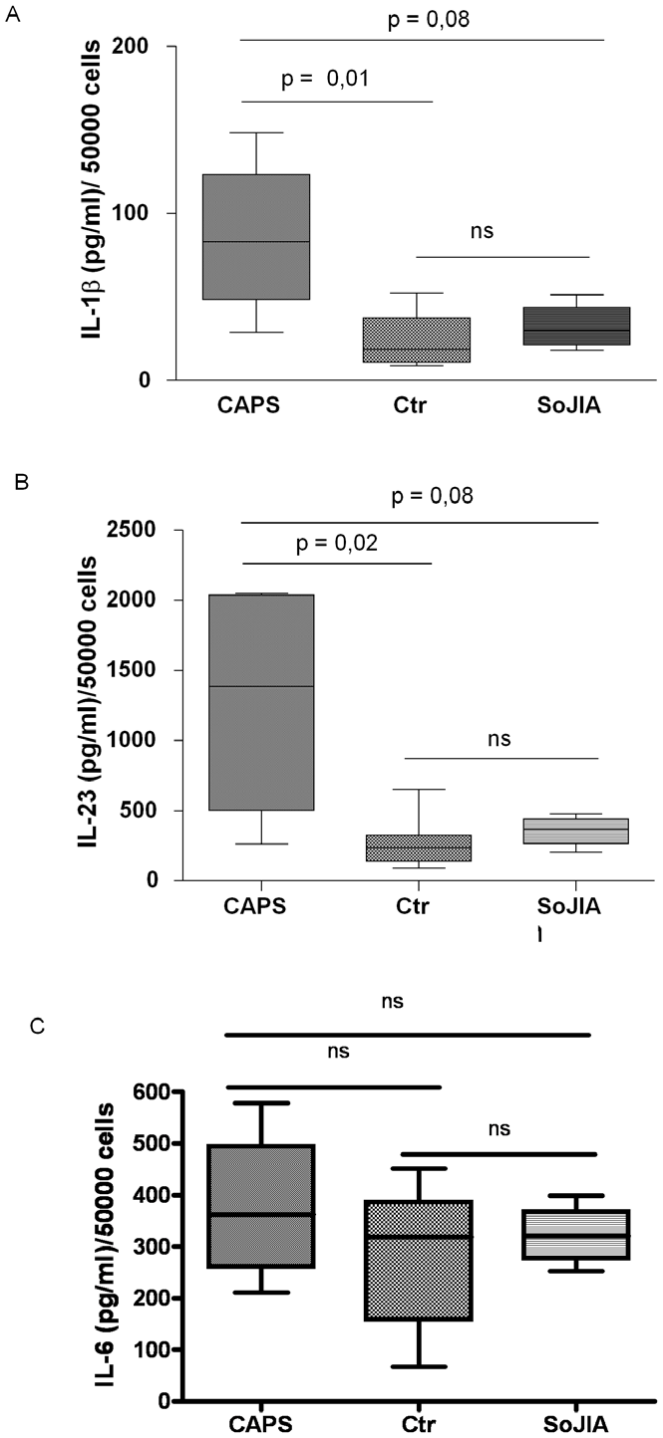
elevated level of IL-1β and IL-23 secreted by MoDCs of CAPS patients upon Zymosan stimulation. Secretion of IL-1β (A), IL-23 (B) and IL-6 (C) by MoDs upon 48 hours of challenge with or without Zymosan in 4 CAPS patients, 10 healthy controls and 4 active SoJIA patients. Bold horizontal lines represent median values. Boxes contain the 50% of values falling between the 25th and 75th percentiles, whiskers lines that extend from the boxes represent the highest and lowest values for each subgroups. Statistical analysis was performed using non-parametric U Mann-Whitney test.

As shown in Figure 4, MoDc from 4 CAPS patients produced significantly higher amount of IL-1β and IL-23 compared to 10 healthy controls and 4 SoJIA patients, whereas no relevant differences were observed in IL-6 secretion among the three subgroups.

Remarkably, after 7 days of treatment with IL-1 blockers, in all CAPS patients we observed a marked reduction of both IL-1β (pre-treatment median 83 ng/ml, range 28.5–148; post-treatment median 24.2, range 20–104) and IL-23 (pre-treatment median 1348.6 ng/ml, range 261–2048; post-treatment median 459.4, range 106–1038) secretion by MoDCs (data not shown).

## Discussion

Given the growing evidence supporting the central role of the IL-1β in the commitment of IL-17 producing cells [6], in the present study we analyzed the IL-23/IL-17 axis in CAPS patients in order to explore a possible involvement of IL-1β in T_H_-17 differentiation in this context.

The protein mutated in CAPS, namely Cryopyrin is a key protein of the inflammasome, a multi-protein complex responsible for activation of the IL-1 converting enzyme (ICE) (or Caspase-1), which in turn converts pro-IL-1β to the mature, active 17 kDa form [28]. In response to a broad range of stimuli, cryopyrin oligomerizes and binds the adaptor protein ASC (Apoptosis associated Speck-like protein containing a CARD). This association activates directly two molecules of Caspase-1 which, in turn, convert pro-IL-1β to the mature, active 17 kDa form. Therefore, activated cryopyrin induces the release of the active form of IL-1β [14], [28], [29].

*NLPR3* mutations are associated with its gain of function with a consequent excessive production of IL-1β [14], [30] even in absence of a second signal, such as extracellular adenosine triphosphate (ATP) [17]. Due to the pleiotrophic proinflammatory effect of IL-1β, the specific inhibition of IL-1β is able to dramatically dampen the severe systemic inflammatory picture of CAPS patients [15], [16].

In contrast to what previously observed in mice models [10], [11], studies in humans have pointed out the potential pivotal role of IL-1β, together with IL-23 and IL-6, in the differentiation of human naïve T cells into IL-17 producing cells [4], [6], [7]. This hypothesis has been disputed by other authors supporting the crucial role of TGF-β in the differentiation of T_H_-17 in humans [31].

In this scenario CAPS, as the prototype of an IL-1 driven human monogenic diseases, represents a powerful tool to investigate the possible involvement of IL-1β in the commitment of naïve T cells towards IL-17 producing cells during the course of chronic inflammatory diseases.

In the present study, several evidences suggest the existence of a T_H_17 skewed phenotype in *NLPR3-mutated* CAPS patients: i) CAPS patients showed increased levels of serum IL-17, ii) stimulated PBMCs displayed higher frequency of IL-17 producing cells compared to healthy controls; iii) monocytes-derived dendritic cells from CAPS patients displayed increased production of IL-1β and IL-23. Notably, either IL-17 serum concentrations or the increased number of IL-17 producing cells observed in CAPS patients were clearly down-modulated by anti IL-1β treatment, suggesting a possible IL-1 dependency of the T_H_17 skewed phenotype observed in CAPS patients. Moreover, when monocyte-derived DCs from the same CAPS patients were evaluated 1 week after anti-IL-1β treatment, a clear reduction of the secretion of both IL-1β and IL-23 was found, as already observed for IL-1β in *NLRP3*-mutated monocytes after *in vivo* treatment with IL-1 blockers [17].

The inhibitory role of anti-IL-1 treatment in the T_H_17 differentiation has been already observed in previous studies. The *in vitro* use of IL-1ra was able to decrease the amount of IL-17 secreted by naïve T cells polarized with the supernatants of MoDcs upon 48 h of Zymosan stimulation [32]. Moreover, PBMCs from celiac patients showed an evident down-modulation of IL-23 in presence of IL-1Ra [33]. This latter observation, supports the hypothesis that the over-expression of IL-23 observed in CAPS patients could be related to an IL-1β dependent mechanism, likely associated to the activation of the inflammasome. In our study the pattern of IL-6 secretion by MoDcs, another cytokine which is classically considered to be crucial to the T_H_17 differentiation, did not show the same behavior observed for IL-1β and IL-23. Even if IL-6 is considered to be down-stream o IL-1β, its expression is not influenced by the activation of the inflammasome. It is therefore conceivable that, at least in this experimental setting, *NLRP3*-mutated MoDC's did not show an over secretion of IL-6, as also observed in circulating monocytes from CAPS patients (Carta et al., manuscript in preparation).

Our study performed in a genetically driven IL-1β mediated disease showed that, at least in CAPS, IL-1β and IL-23 may play the major role in the differentiation of T_H_17 cells.

The different behavior observed in an other systemic inflammatory disease such as SoJIA, let us make the hypothesis that the above mentioned findings are likely secondary to the genetic defect of the *NLRP3* gene rather than to a non-specific influence of the ongoing inflammation.

SoJIA is a multifactorial, inflammatory disease that share a number of clinical features (systemic inflammation, arthritis, rash, persistent elevation of acute phase reactants) with CAPS. The pathogenesis of SoJIA is still rather controversial even taking into account the likely heterogeneity of this condition. A number of experimental evidences supports the pivotal pathogenic role of IL-6 in SoJIA [34]–[37]. These findings are in line with the efficacy of IL-6 blockade observed in this condition [38], [39].

However, as observed in other IL-6 mediated disease such as Castelman's disease and smoldering multiple myeloma [40], [41], a variable percentage of these patients (from 40 to 87% according to the different studies) also shows a dramatic and persistent response to anti-IL-1 blockade, with the rest of SoJIA patients being resistant to such a treatment [23], [42], [43],

The different rate of response to anti IL-1 treatment observed in SoJIA patients may explain the variability of the findings concerning the actual pathogenic role of IL-1 in this latter disease. In some studies, gene expression analysis revealed the presence of a prevalent IL-1β signature in SoJIA [42], [44]. The same picture was not found in other independent studies [45]–[47], that however confirmed the prevalent role of genes of innate immunity in this condition. Similar variability was also observed when the pattern of secretion of IL-1β from SoJIA PBMCs and/or monocytes was compared to healthy controls [23], [42], [48]. In any case, when compared to active *NLRP3*-mutated CAPS patients, monocytes from active SoJIA secrete lower amounts of IL-1β [17], [23] and displayed a different kinetics of secretion [49], independently from the pattern of clinical response to IL-1β blockade [23]. These latter observations do not exclude the possibility that in SoJIA the paracrine secretion of IL-1β in privileged sites can induces different target cells to produce large amounts of IL-6, thus explaining the good response to IL-1 blockade, as observed in other “IL-6-driven” diseases [40], [41], [50].

In the present study the serum concentrations of IL-6 in CAPS patients, that we found similar to those reported by Goldbach-Mansky et al. [16], were significantly lower when compared to active SoJIA patients. This finding is in agreement with the normal secretion of IL-6 observed in CAPS MoDc's and monocytes (Carta et al. manuscript in preparation) and may explain the anecdotal poor response to IL-6 blockade in CAPS patients [51].

In the present study, SoJIA patients showed a less evident elevation of IL-17 during the active phase of disease and no significant increase in the frequency of IL-17 producing cells. Our observation do not exclude *per se* the possible contribution of the IL-17/IL-23 axis in.

SoJIA. Indeed, as already observed in RA and other forms of JIA [5], [52], SoJIA patients display an increased frequency of IL-17 producing cells in synovial fluid when compared to peripheral blood (not shown). The finding of a T_H_17 skewed phenotype in a pure IL-1 driven disease such as CAPS further confirms the pivotal role of IL-1β in the T_H_17 differentiation in human inflammatory diseases. Interestingly, recent evidences coming from different studies have pointed out the pivotal role of IL-1β in the *in vivo* and *in vitro* differentiation of naïve T cells into TH-17 cells also in mice [24], [53], [54], disputing the existence of a actual dichotomy between mice and humans [54]. Even if CAPS represents the example of an inflammatory diseases predominantly driven by a primary deregulation of the innate arm of the immune response, our study illustrate its possible consequences on the adaptive response. Whether the expansion of T_H_17 phenotype observed in CAPS patients is simply an epiphenomenon secondary to the over-secretion of IL-1β due to *NLPR3* mutations or it may contribute to the maintenance of chronic inflammation, likely through the influence of IL-17 in neutrophils mobilization and recruitment, is still unclear.

Two recent parallel studies using *NLRP3* mutant knock-in mouse strains were not able to clarify this issue. In fact, the *NLPR3* A352V and L353P *knockin* mouse were characterized by an early lethality and by a minimal involvement of T cells and IL-17 and IL-22 expression in either spleen and skin infiltrate [55]. Conversely, the generation of the *NLPR3*-R258W mice was associated with a more prolonged survival and with the development of a spontaneous skin inflammation with a prevalent neutrophilic infiltration, but with an evident lymphoid component that was characterized by a T_H_17 cytokine predominance [56].

In conclusion, the present study performed in a genetically driven IL-1β mediated disease supports the pivotal role of IL-1β in T_H_17 differentiation in humans, and suggest the hypothesis that a continuous cross-talk between innate and adaptive immunity may contribute to the development and maintenance of chronic inflammation in these conditions.
